# Vulnerability in elderly patients with gastrointestinal cancer – translation, cultural adaptation and validation of the European Portuguese version of the Vulnerable Elders Survey (VES-13)

**DOI:** 10.1186/s12885-015-1739-2

**Published:** 2015-10-16

**Authors:** F. Carneiro, N. Sousa, LF Azevedo, D. Saliba

**Affiliations:** 1 Department of Medical Oncology, Instituto Português de Oncologia do Porto, Rua Dr. António Bernardino de Almeida, Porto, 4200-072 Portugal; 2 Department of Health Information and Decision Sciences (CIDES) and Center for Research in Health Technologies and Information Systems (CINTESIS), Faculty of Medicine, University of Porto, Porto, Portugal; 3 Faculdade de Medicina da Universidade do Porto (CIM - FMUP), Rua Dr. Plácido da Costa, s/n, Porto, 4200-450 Portugal; 4 The University of Los Angeles Borun Center, The VA Greater Los Angeles GRECC and RAND Santa Monica, 10945 Le Conte Avenue, Suite 2339, Los Angeles, CA 90095 USA

**Keywords:** VES-13, Vulnerability, Gastro-intestinal cancer

## Abstract

**Background:**

“Vulnerable Elders Survey” (VES-13) is a questionnaire accurate in predicting functional decline and highly correlated with comprehensive geriatric assessment in identifying vulnerable elderly. The purpose of this study was to translate, cultural adapt and validate the first Portuguese cross-cultural version of VES-13 and to estimate the prevalence of vulnerability in Portuguese elderly gastrointestinal (GI) cancer patients.

**Methods:**

VES-13 European Portuguese translation and cultural adaptation was developed according to internationally accepted guidelines. Test-retest reliability and internal consistency were assessed by calculating the Kappa statistic and by analyzing the inter-item and item-total correlation matrices and calculation of Cronbach’s alpha coefficients, respectively. Construct and criterion validity was assessed by Spearman’s correlation coefficient between VES-13 and each EQ-5D-5 L dimension, clinical judgment and performance status.

**Results:**

The translated and culturally adapted version of VES-13 revealed high test-retest reliability (test-retest Kappa ≥ 0.612; p < 0.001) in the pilot study (*n* = 22). For the validation phase 206 patients with GI cancer were recruited (median age: 73 years; colo-rectal cancer: 63 %). Criterion validity was confirmed by adequate correlations between VES-13 and clinical judgment of vulnerability, ECOG and KPS scores. Construct validity was confirmed by moderate correlations with most of EQ-5D-5 L dimensions. Cronbach’s alpha of the questionnaire was 0.848.

The estimated prevalence of vulnerability is 50 % (CI95% 0.43-0.56).

**Conclusions:**

The European Portuguese version of VES-13 is a valid and reliable approach to screening elderly cancer patients for geriatric needs. In our setting, one in two elderly patients was likely to be vulnerable or frail which stresses the importance of their correct identification to better inform cancer management.

**Electronic supplementary material:**

The online version of this article (doi:10.1186/s12885-015-1739-2) contains supplementary material, which is available to authorized users.

## Background

As the western population ages overall cancer burden will increase [1]. Cancer of the digestive tract accounts for 30 % of new cancer cases per year and 60 % of these are diagnosed in patients older than 65 years [1]. In Portugal, gastro-intestinal (GI) cancer is the most incident cancer and approximately 10,000 new cases per year are diagnosed in patients ≥ 65 years old [2].

Elderly cancer patients are a heterogeneous population. They are more likely to present multiple co-morbid conditions and are more frequently affected by polypharmacy, depression and cognitive impairment than younger individuals [3–8]. Moreover, because this population is frequently under-represented in clinical trials the effectiveness and toxicity profile of standard treatment protocols are less well established for the elderly [4]. Both issues increase uncertainty when therapeutic decisions have to be made [4].

The higher inter-individual variability of the elderly led geriatric medicine to establish the concept of vulnerability which attempts to describe patients with increased susceptibility to adverse outcomes [7]. In geriatric oncology, vulnerability is also associated with prognosis [9-11].

The best way to identify vulnerability is through a biopsychosocial evaluation commonly known as comprehensive geriatric assessment (CGA) [6, 12].But a full CGA is time and human resource consuming, making its incorporation into current clinical practice less feasible [6]. The “Vulnerable Elders Survey” (VES-13), a 13-item self-report questionnaire, distinguishes fit elders from the frail or vulnerable ones. This tool has been shown to identify elderly patients who would require a comprehensive geriatric evaluation [13–17]. However, no validated translation to European Portuguese was available.

Our primary goal was to translate, culturally adapt and validate the VES-13 questionnaire for the Portuguese population. The secondary research objective was to estimate the prevalence of vulnerability in elderly patients with GI neoplasms in Portugal.

## Methods

### Translation and face validity

The authors followed the European Organization for Research and Treatment of Cancer (EORTC) guidelines - Quality of Life Group Translation Procedure; and Guillermin et al. recommendations [18, 19]. Briefly, the original questionnaire was translated into European Portuguese and culturally adapted by two healthcare professionals with English fluency, knowledgeable of the translation purpose. This draft version was translated back into English, by two English translators, and compared to the original questionnaire by the investigators and the original VES-13 authors, to assess comprehension of the applied concepts and wording. No problems were identified at this stage.

Face validity of the translated questionnaire was assessed by six medical oncologists at our GI Cancer Clinic. They were asked to review the original and translated questionnaires and classify each question, according to comprehension and accuracy of the translation, using a numerical rating scale of 10 points (1 - poorly clear, to 10 - completely clear).

### Patient recruitment

Cancer patients admitted at our Comprehensive Cancer Centre age ≥65 years with histologically confirmed GI Cancer, Portuguese fluency, and no history of previous systemic therapy for cancer were eligible for both the pilot and prospective validation cohort. Patients presenting cognitive impairment, confusional syndrome or who were illiterate or foreign individuals were excluded from the pilot study. The pilot study also excluded patients unable to read.

This work has been approved by the ethical committee of the “Instituto Português de Oncologia do Porto” in Portugal, institution where it was developed and all the subjects gave their informed consent.

### Pilot study: cultural adaptation and test-retest reliability

The questionnaire was applied by one of the investigators to included consecutive patients (first pilot *n* = 20, second pilot *n* = 22) who were asked to rate each question for comprehension using the previously described numerical rating scale of 10 points. Each patient completed the VES-13 questionnaire twice within 1 to 30 days. At this point, a question was to be reviewed if it had a single rating ≤5 (corresponding to reasonably clear), or if any comprehension problem was noted by the interviewer. Concerns regarding question 3f made necessary a second pilot, after questionnaire adaptation.

### Prospective cohort study: construct and criterion validity

After completion of the pilot study, the European Portuguese version of VES-13 was prospectively applied to a cohort of 200 patients to assess internal consistency and construct and criterion validity [20–22]. To assess construct validity we selected EQ-5D-5L as comparator [23]. EQ-5D-5L is a generic health related quality of life questionnaire which includes five dimensions and a visual analogue scale (VAS) assessing general health. Each dimension is recorded in five severity levels (no problems, slight, moderate, severe and extreme problems, graded from 1 to 5, respectively). The VAS records an individual’s rating for their current health-related quality of life (ranging from 0 - worst imaginable health, to 100 – best imaginable health). Predefined hypothesis about relationships among dimensions of EQ-5D-5L and VES-13 were tested to assess construct validity. To assess criterion validity we used the clinical impression of a trained medical oncologist, blinded to the responses on VES-13, regarding patient’s vulnerability and performance status (PS). Each medical oncologist was instructed to consider the Eastern Cooperative Oncology Group classification – ECOG [24] and Karnofsky scale – KPS [25], and to categorize each patient into the following groups: fit, vulnerable or frail. Performance status was estimated according to exact ECOG PS and KPS scales definition (ECOG PS ranging from 0 –able to carry on all pre-disease performance without restriction, to 5 – dead; KPS ranging from 100 – normal, no complaints, to 0 – dead). Correlations among these criteria and VES-13 were evaluated to assess criterion validity.

### Statistical analysis

Patient’s demographics and clinical characteristics were studied using descriptive statistics as appropriate. Numerical variables were described with means and standard deviation or with medians and interquartile ranges, depending on the asymmetry of their distributions. Categorical variables were described as absolute and relative (percentages) frequencies. Performance status was categorized as follows: ECOG ≤1 and ≥2 and KPS 100–80 and ≤70. Charlson comorbidity index (CCI) was used to estimate the burden of co-morbid conditions. When testing hypothesis about continuous variables, Student’s *t*-tests were used to compare two groups when normality assumptions were confirmed and Mann–Whitney U tests were used if normality could not be assumed). When testing hypothesis about categorical variables, Chi-square test and Fisher’s exact test were used as appropriate.

The test-retest reliability of the Portuguese version of VES-13 was assessed in the pilot study by calculating the Kappa statistic for each item to assess agreement between test and retest scores [26]. This index takes values between −1 and 1; values near 1 show high test-retest reliability. The categorization by Landis and Koch was used for interpretation of κ values (<0.00 – no agreement; 0.01-0.20 – slight; 0.21-0.40 – fair; 0.41-0.60 – moderate; 0.61-0.80 – substantial and 0.81-1.00 – almost perfect agreement) [27]. Additionally, we calculated the test-retest reliability coefficient (correlation coefficient) for VES-13 total scale score.

Internal consistency of translated VES-13 items was explored by analyzing the inter-item and item-total correlation matrices and calculation of Cronbach’s alpha coefficients. This coefficient ranges from 0 to 1, and larger values indicate higher internal consistency. As recommended by Nunnally and Bernstein, alphas ≥0.70 were considered adequate [21]. An estimation of Cronbach’s alpha if an item were to be deleted from the scale was used to identify which items affected the questionnaire’s internal consistency the most.

Construct and criterion validity was assessed by calculating Spearman’s correlation coefficient between VES-13 and each EQ-5D-5L dimension, clinical judgment and performance status. Interpretation of correlation coefficients was based on the quantitative criteria and qualitative descriptors defined by Cohen [28] (low correlations for coefficients with absolute value between 0.10 and 0.29; moderate correlations for coefficients between 0.30 and 0.49 and high correlations for coefficients between 0.50 and 1.00).

Exploratory factor analysis for VES 13 European Portuguese version was performed using principal components analysis for factor extraction. The hypothesis of unidimensionality of VES-13 was assessed. Selection of the number of factors to retain took into account Kaiser’s criterion (eigenvalues larger than one); graphical analysis of the Scree-plot; and the total variance explained. If adequate, to improve interpretation of factors, orthogonal varimax rotations were to be applied. The Kaiser-Meyer-Olkin (KMO) measure and the Bartlett’s test of sphericity were assessed.

Finally, we performed a ROC curve analysis, to assess the best cutoff point for VES-13 total score for discrimination of Frail/Vulnerable elders, assuming the attending physician’s clinical judgment as the gold standard. Best cutoff selection criterion was based on the method of minimization of the distance to the left upper corner of the ROC plot, calculated as √(1-Sn)^2^ + (1-Sp)^2^.

A prospective cohort of 200 consecutively enrolled senior GI cancer patients (≥65 years), would allow an estimation of the prevalence of vulnerability/frailty with a 95 % confidence level margin of error of 0.07. This sample size would also allow an estimation of validity coefficients (correlation coefficients) larger than 0.20, with 95 % confidence level and 90 % power.

Statistical analysis was performed using the Statistical Package for the Social Sciences Version 20.0 for Windows (SPSS®). Whenever statistical hypothesis testing was used, a significance level of α = 5 % was considered.

## Results

### Translation and cultural adaptation

After translation and cultural adaptation, all questions scored 6 or higher, corresponding to reasonable comprehension during face validation and no changes were deemed necessary.

The pilot study included 20 patients and comprehension difficulties were apparent to the interviewer for male patients answering two questions that included examples of household tasks. These problems were discussed with the original VES-13 authors, and the Portuguese questionnaire was adapted with the inclusion of different domestic tasks examples. A second pilot test was implemented with 22 patients and no difficulties were noted. All questions scored 6 or higher in VAS and test-retest reliability revealed substantial to perfect agreement between test and retest for individual items (test-retest Kappa ranging from 0.612 to 1.000, *p* < 0.001) and very high correlation between test and retest VES-13 total scale scores, as shown in Table 1 [27].

**Table 1:** Intra-individual classification and reliability of each VES-13

VES-13 question	VAS for comprehension		Test-retest reliability	
	median [interquartile range]		Reliability coefficients (p)	
Item 1	8.0	[8–9]	1.000	(<0.001)
Item 2	8.0	[8–9]	0.736	(<0.001)
Item 3a	8.0	[8–9.25]	0.771	(<0.001)
Item 3b	8.0	[8–9.25]	0.612	(<0.001)
Item 3c	8.0	[8–9.25]	0.906	(<0.001)
Item 3d	8.0	[8–9.25]	0.792	(<0.001)
Item 3e	8.5	[8–9.25]	0.938	(<0.001)
Item 3f	8.5	[8–9.25]	0.823	(<0.001)
Item 4a	8.5	[8–9.25]	0.911	(<0.001)
Item 4b	8.5	[8–9.25]	1.000	(<0.001)
Item 4c	8.5	[8–9.25]	0.831	(<0.001)
Item 4d	8.5	[8–9.25]	1.000	(<0.001)
Item 4e	8.5	[8–9.25]	1.000	(<0.001)
VES-13 Total Score		-	0.924	(<0.001)

VES-13 – Vulnerable Elders Survey; VAS – Visual Analogue Scale; p – significance level

Reliability coefficients – Kappa statistic measuring agreement between test and retest individual items and Pearson’s correlation coefficient measuring reliability between test and retest VES-13 total scale scores

### Internal consistency and construct and criterion validity

**Fig. 1:**
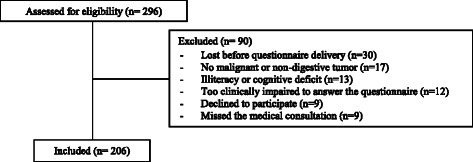
Flowchart of validation study selection process

The VES-13, EQ-5D-5L and medical oncologist’s clinical assessment were applied during 6 months (June to November, 2012). During this period, 296 elderly patients with GI Cancer were admitted to our GI Cancer Clinic and a total of 206 patients were included (Fig. 1 describes the selection process and reasons for exclusion). Demographic and clinical characteristics of the cohort are described in Table 2. The 90 individuals not included in the sample had epidemiological and clinical characteristics similar to those included in the study.

**Table 2:** Clinical and demographic characteristics

	VES-13 < 3		VES-13 ≥ 3		Total	
	*n* = 104	(%)	*n* = 102	(%)	*n* = 206	(%)
Male	77	(74)	56	(55)	133	(65)
Age - years, median [range]	72	[65–84]	77	[65–89]	73	[65–89]
Charlson comorbidity index, median [range]	6	[5–15]	8	[5–15]	7	[5–15]
Diabetes mellitus	29	(28)	33	(32)	62	(30)
Cardiovascular disease	12	(12)	24	(24)	36	(17)
No. concomitant drugs, median [range]	3	[1–9]	4	[1–8]	3	[1–9]
Primary cancer topography						
Colorectal	66	(64)	64	(63)	130	(63)
Gastro-esophageal	29	(28)	24	(24)	53	(26)
Pancreas	3	(3)	10	(10)	13	(6)
Cancer stage (AJCC 7th edition)						
I	9	(9)	8	(8)	17	(8)
II	23	(22)	20	(20)	43	(21)
III	43	(42)	34	(33)	77	(37)
IV	22	(21)	34	(33)	56	(27)

VES-13 – Vulnerable Elders Survey: < 3 → fit; ≥ 3 → vulnerable/fragile; AJCC – American Joint Committee of Cancer

The cohort’s median age was 73 years (29 % ≥80 years) and 65 % of the subjects were male. Colorectal cancer was the most frequent tumor location (63 %) and 3 % of patients had a history of previous neoplasms. The most prevalent co-morbidities were diabetes (*n* = 62, 30 %) and cardiovascular disorders (*n* = 36, 17 %). The median CCI was 7 (interquartile range: 6–11). Fifty-eight patients (28 %) were taking five or more daily drugs, and the more frequent therapeutic groups, as defined by the World Health Organization Anatomical Therapeutic Chemical/Defined Daily Dose, were “cardiovascular system” and “alimentary tract and metabolism” drugs.

Compliance with VES-13 and EQ-5D-5L questionnaires was 100 %, except for EQ-5D-5L questions “pain/discomfort”, “anxiety/depression” and VAS scale, which were above 98 %. Summary results for quality of life assessed using EQ-5D-5L are presented in Table 3. A proportion greater than 70 % of patients indicated that they were facing no problems or slight problems in all EQ-5D-5L dimensions; “self-care” presented the highest result with over 80 % of patients experiencing no problems or slight problems. Overall quality of life assessment for the cohort revealed a median EQ-VAS score of 60 percent (interquartile range: 50–75).

**Table 3:** Functional status and quality of life

	VES-13 < 3		VES-13 ≥ 3		*P* value *
	*n* = 104	(%)	*n* = 102	(%)	
EQ-5D-5L					
Mobility					<0.001
No problems	72	(69)	16	(16)	
Problems	32	(31)	86	(84)	
Self-care					<0.001
No problems	97	(93)	35	(34)	
Problems	7	(7)	67	(66)	
Usual activities					<0.001
No Problems	84	(81)	20	(20)	
Problems	20	(19)	82	(80)	
Pain/discomfort					0.001
No problems	48	(46)	24	(24)	
Problems	55	(54)	77	(76)	
Anxiety/depression					0.193
No problems	34	(33)	25	(24)	
Problems	69	(67)	76	(76)	
VAS, median [p25-p75]	70	[60–80]	50	[40–60]	<0.001**
CLINICAL JUDGMENT					<0.001
Fit	85	(82)	33	(32)	
Vulnerable/fragile	19	(8)	69	(68)	
ECOG PERFORMANCE STATUS					<0.001
0	58	(56)	13	(13)	
1	46	(44)	38	(37)	
≥2	-		51	(50)	
KARNOFSKY PERFORMANCE STATUS					<0.001
100	28	(27)	6	(6)	
90	48	(46)	17	(17)	
80	26	(25)	29	(28)	
≤70	2	(2)	50	(49)	

VES-13 – Vulnerable Elders Survey: < 3 → fit; ≥ 3 → vulnerable/fragile; VAS - Visual Analogue Scale; ECOG – Eastern Cooperative Oncology Group;* - Chi-square test; p - significance level; **Mann–Whitney test

**Fig. 2:**
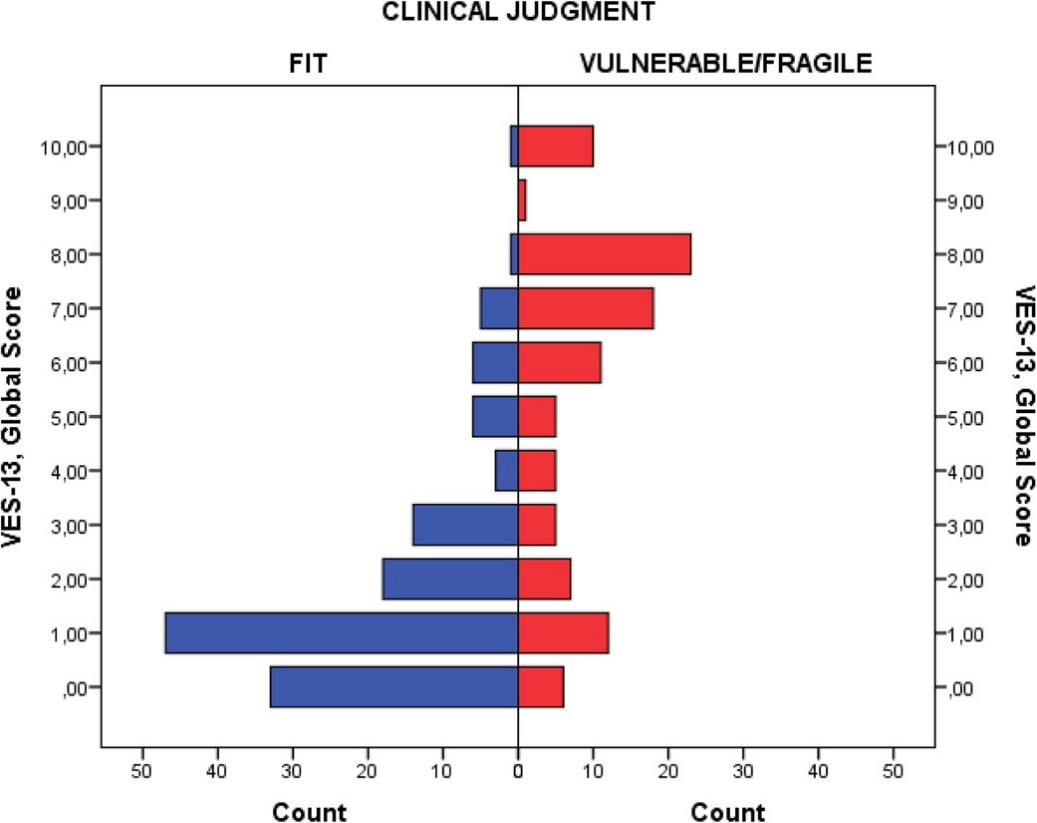
Distribution of VES-13 global score for the Fit and Vulnerable/Fragile elders, as classified by the clinical judgment of the attending physician

**Fig. 3:**
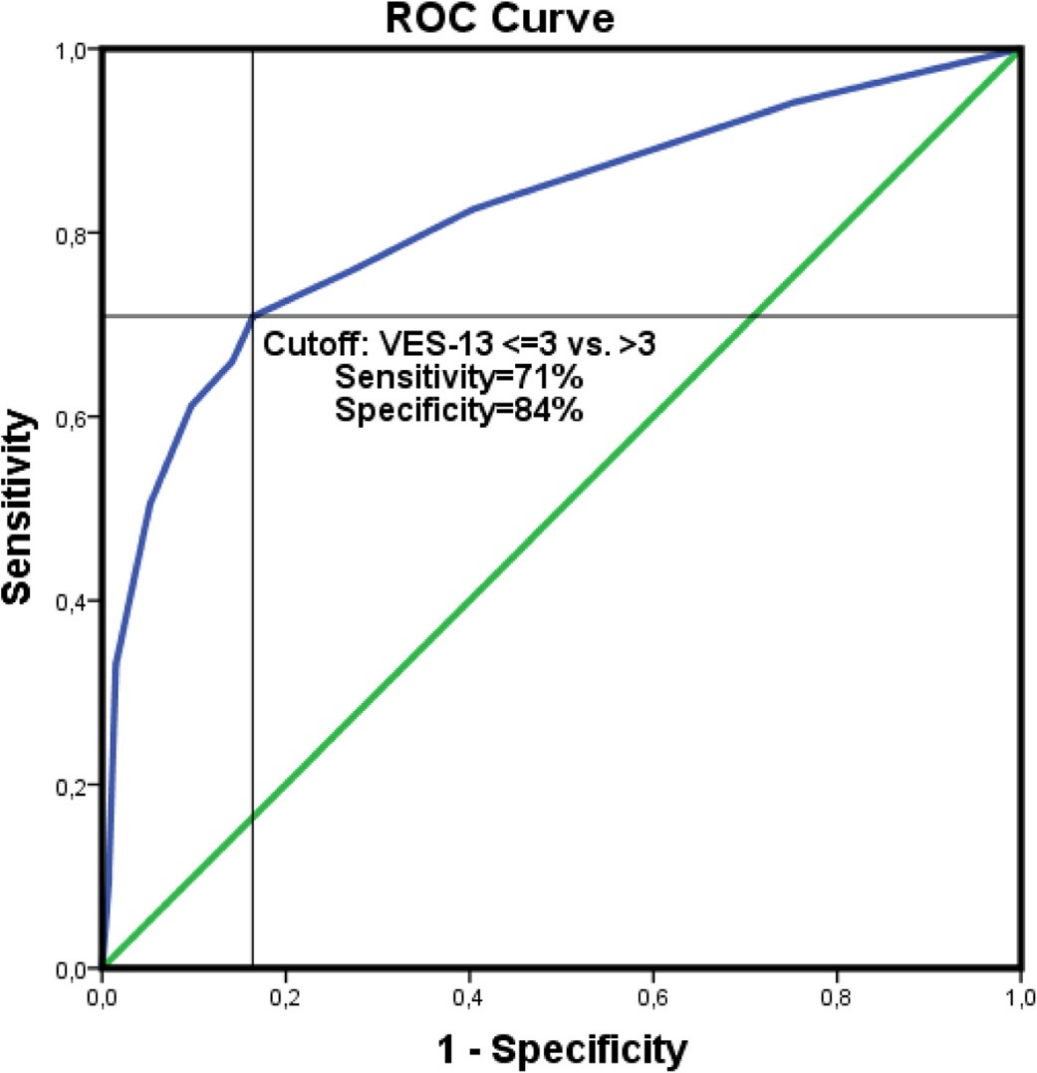
ROC curve analysis for the VES-13 total score. Legend : ROC curve analysis for the VES-13 total score, assuming the clinical judgment of the attending physician as the gold standard, showing the cutoff value of >3 as the most appropriate for maximizing both sensitivity (71 %) and specificity (84 %). The area under the ROC curve was C = 0.818 (95 % CI [0.762 – 0.875])

VES-13 European Portuguese version of the questionnaire showed high internal consistency, with Cronbach’s alpha if item deleted ranging from 0.826 to 0.880, and a Cronbach’s alpha for the scale score of 0.848, Table 4. When assessing the correlation of VES-13 and EQ-5D-5L dimensions we obtained, as expected, higher correlation scores for “mobility”, “self-care” and “usual activities” (r_s_: 0.634, 0.625 and 0.652 respectively). Although not so strong, statistically significant correlations with “pain/discomfort” (r_s_: 0.329) and “anxiety/depression” (r_s_: 0.178) domains and with VAS scale (r_s_: −0,527) were also observed. There were moderate to strong correlations between VES-13 and clinical judgment, ECOG and KPS scales (r_s_: −0.499, 0.599, and −0.576, respectively). Table 5 presents the summary statistics for the correlation coefficients between VES-13 and EQ-5D-5L, performance status and clinical impression. In Fig. 2 we can also see and contrast the distribution of VES-13 total score for the fit and vulnerable/fragile elders, as classified by the clinical judgment of the attending physician. In Fig. 3 we present a ROC curve analysis of the VES-13 total score, assuming the clinical judgment of the attending physician as the gold standard; and showing the cutoff value of >3 as the most appropriate for maximizing both sensitivity and specificity. For this cutoff value the sensitivity was 71 % and the specificity was 84 %. The estimate of the area under the ROC curve was C = 0.818 (95 % CI [0.762 – 0.875]).

**Table 4:** VES-13 internal consistency

VES-13 question	Classification (points)	n	(%)	Cronbach’s alpha if item deleted
Item 1	0	119	(58)	0.880
	1	68	(33)	
	3	19	(9)	
Item 2	0	61	(30)	0.849
	1	145	(70)	
Item 3a	0	155	(75)	0.829
	1	51	(25)	
Item 3b	0	162	(79)	0.829
	1	44	(21)	
Item 3c	0	184	(89)	0.835
	1	22	(11)	
Item 3d	0	194	(94)	0.847
	1	12	(6)	
Item 3e	0	164	(80)	0.829
	1	42	(20)	
Item 3f	0	118	(57)	0.826
	1	88	(43)	
Item 4a	0	156	(76)	0.827
	4	50	(24)	
Item 4b	0	178	(86)	0.835
	4	28	(14)	
Item 4c	0	189	(92)	0.836
	4	17	(8)	
Item 4d	0	165	(80)	0.833
	4	41	(20)	
Item 4e	0	155	(75)	0.829
	4	51	(25)	
VES-13 TOTAL SCORE	-	-	-	0.848

**Table 5:** Criterion and construct validity

	VES-13	
	rs	(*p* value)
EQ-5D-5L		
Mobility	0.688	(<0.001)
Self-care	0.690	(<0.001)
Usual activities	0.732	(<0.001)
Pain/discomfort	0.405	(<0.001)
Anxiety/depression	0.237	(0.001)
VAS (mean)	−0.592	(<0.001)
CLINICAL JUDGMENT	−0.570	(<0.001)
ECOG PS	0.614	(<0.001)
KPS	−0.622	(<0.001)

Exploratory factor analysis, with factor extraction using principal components, was performed for VES-13. The factor solution included a first component with eigenvalue 6.41 and 49.3 % of the variance explained, a second component with eigenvalue 1.08 and 8.3 % of the variance explained, and all other components with eigenvalues lower than 1.00 and smaller percentages of variance explained. Based on the analysis of the scree plot, the eigenvalues of the components and the percentage of the variance explained, the one factor solution was clearly the more appropriate, supporting the hypothesis of unidimensonality of VES-13. Although the strict application of the Kaiser rule would imply the selection of a two factor solution, the fact is that the second component had an eigenvalue marginally above 1.00 and a low percentage of variance explained, thus a one factor solution is clearly a more sensible solution in this case.

Loadings found in the one factor solution and the KMO and Bartlett’s test of sphericity are presented in Tables 6 and 7. It is possible to assess the adequacy of the one factor solution by observing that loadings of most items are above 0.6, with only the first item (Age category) having a loading of 0.375; and taking into consideration the high value of the KMO statistic (KMO = 0.905) and the result of the Bartlett’s test (*p* < 0.001).

**Table 6:** Exploratory factor analysis (a) for the Portuguese version of VES-13

VES-13 question	Factor loadings
	One Factor Solution
(a) Exploratory factor analysis	
Item 1	0.375
Item 2	0.616
Item 3a	0.792
Item 3b	0.800
Item 3c	0.768
Item 3d	0.752
Item 3e	0.788
Item 3f	0.797
Item 4a	0.711
Item 4b	0.556
Item 4c	0.656
Item 4d	0.655
Item 4e	0.731

VES-13 – Vulnerable Elders Survey. Eigenvalue and percentage of variance explained for the one factor solution were 6.405 and 49.27 %, respectively. KMO statistic was 0.905 and Bartlett’s test of sphericity had *p* < 0.001

**Table 7:** Inter-item correlation matrix (b) for the Portuguese version of VES-13

VES-13 question	Item 1	Item 2	Item 3a	Item 3b	Item 3c	Item 3d	Item 3e	Item 3f	Item 4a	Item 4b	Item 4c	Item 4d	Item 4e
(b) Inter-item correlation matrix													
Item 1	1.000	0.058	0.293	0.241	0.196	0.173	0.309	0.279	0.239	0.312	0.192	0.233	0.271
Item 2	0.058	1.000	0.385	0.461	0.387	0.440	0.483	0.523	0.398	0.262	0.406	0.418	0.326
Item 3a	0.293	0.385	1.000	0.674	0.677	0.597	0.614	0.607	0.443	0.312	0.462	0.432	0.525
Item 3b	0.241	0.461	0.674	1.000	0.676	0.561	0.592	0.685	0.487	0.344	0.477	0.421	0.461
Item 3c	0.196	0.387	0.677	0.676	1.000	0.616	0.498	0.512	0.502	0.371	0.452	0.456	0.466
Item 3d	0.173	0.440	0.597	0.561	0.616	1.000	0.560	0.504	0.466	0.369	0.489	0.358	0.550
Item 3e	0.309	0.483	0.614	0.592	0.498	0.560	1.000	0.714	0.454	0.298	0.478	0.504	0.519
Item 3f	0.279	0.523	0.607	0.685	0.512	0.504	0.714	1.000	0.493	0.346	0.367	0.472	0.580
Item 4a	0.239	0.398	0.443	0.487	0.502	0.466	0.454	0.493	1.000	0.563	0.436	0.497	0.489
Item 4b	0.312	0.262	0.312	0.344	0.371	0.369	0.298	0.346	0.563	1.000	0.357	0.292	0.415
Item 4c	0.192	0.406	0.462	0.477	0.452	0.489	0.478	0.367	0.436	0.357	1.000	0.360	0.478
Item 4d	0.233	0.418	0.432	0.421	0.456	0.358	0.504	0.472	0.497	0.292	0.360	1.000	0.505
Item 4e	0.271	0.326	0.525	0.461	0.466	0.550	0.519	0.580	0.489	0.415	0.478	0.505	1.000

VES-13 – Vulnerable Elders Survey. Eigenvalue and percentage of variance explained for the one factor solution were 6.405 and 49.27 %, respectively. KMO statistic was 0.905 and Bartlett’s test of sphericity had *p* < 0.001

### Prevalence of vulnerability in elderly patients with GI cancer

The proportion of vulnerable elderly GI cancer patients was 0.50 (CI95%: 0.43-0.56). Vulnerable patients were more likely to have higher EQ-5D-5L scores on every dimension, meaning higher prevalence and magnitude of problems, and lower EQ-VAS values, meaning a perception of worse quality of life. These patients also had worse performance status (higher mean ECOG-PS and lower KPS). Vulnerable patients had higher CCI scores (*p* < 0.001) and were also more likely to have higher polypharmacy levels (32 % versus 19 % of patients were receiving ≥5 daily drugs).

## Discussion

Aging results in physiologic decline and there is consensus that oncologic treatment decisions should be based on a patient’s biologic age rather than his chronologic age [29, 30]. Multiple tools have been developed to identify vulnerability and frailty, but there is no consensus on a single optimal approach. The International Society of Geriatric Oncology (SIOG) considers VES-13 a useful screening tool to identify vulnerable elders [31]. This survey questionnaire predicts impaired functional status but was not available in European Portuguese.

The proposed European Portuguese version presented in this paper was developed according to internationally accepted guidelines [18, 19]. After the translation procedures, pilot studies were performed to assess comprehension difficulties and questionnaire translation adequacy. Test-retest reliability of the questionnaire’s total score and individual items was generally very high, expressed by the high reliability coefficient for the total score and the near 1.0 Kappa values for each individual item. Internal consistency, which ensures the questionnaire delivers consistent and reliable scores was, for each item and globally, high (Cronbach’s alpha of 0.848).

To assess construct validity we used EQ-5D-5L as comparator. This tool includes five different dimensions with predictable relationships with the concept assessed by VES-13. In the present study, in accordance with our *a priori* predictions, a strong correlation was observed between the EQ-5D-5L dimensions of “mobility”, “self-care” and “usual activities” and VES-13. “Pain/discomfort” and “anxiety/depression” dimensions are not directly assessed with VES-13; nonetheless, we found a weak but statistically significant correlation. The EQ-VAS obtained a negative correlation with VES-13 scores because, as opposed to other dimensions, higher VAS values are associated with higher perceived quality of life, thus with less vulnerability. To assess criterion validity we used three different criteria that were assumed to indirectly measure the vulnerability construct - clinical judgment, ECOG PS and KPS. We used ECOG and KPS even though there is strong criticism in their use on geriatric oncology but these scales, standard measures used in clinical practice, allowed us to make the criterion validity of our instrument.

All were highly correlated with VES-13. In summary, assessment of construct and criterion validity as performed demonstrates the adequate validity of the translated and culturally adapted VES-13 European Portuguese version. However, despite a correct identification of most vulnerable patients, clinical judgment of vulnerability by a trained medical oncologist classified 16 % of patients as fit while VES-13 scored them as vulnerable/frail patients. These results point to utility of the VES-13 as an initial screen to identify who should go on to receive additional comprehensive geriatric assessment before determining their clinical classification.

Construct validity of VES-13 was also explored with exploratory factor analysis, with factor extraction using principal components; and the model described for VES-13 revealed the appropriateness of the one factor solution and the unidimensionality hypothesis. It is interesting to notice that the loadings in the one factor solution for each VES-13 item were very high (generally above 0.6), however for the first item, age category (“below 75 years old”, “between 75–84 years” and “85 years or above”, with higher scores as age increases), we observed a relevantly lower loading of 0.375, indicating that this was the single item with the lowest association and consistency with the VES-13 total score and the vulnerability/frailty construct. This is a very interesting result that underlines the need for careful evaluation of elderly cancer patients; as age, by itself, should not be viewed as the most important factor when assessing an elder as vulnerable/frail.

Internal validity is critical in any research study and this judgment requires awareness of possible biases limiting the study conclusions [32]. Withdrawal bias although moderately high, as evidenced by a loss of 10 % of potentially eligible patients, probably does not invalidate our conclusions. Patients who were lost were as likely to be given anti-cancer treatment as those included and clinical and demographic characteristics were similar between patients lost and those included (data not shown). The main reason for loss of eligible patients was the high clinical pressure on recruiting clinicians at our institution, which mandated an adjustment to patient recruitment half way into the study. The investigators tried to avoid selection bias by establishing precise inclusion and exclusion criteria. Nevertheless 4 % of excluded patients were too sick to answer, and thus fragile, and some of the patients who were not assessed for inclusion might also have contributed to selection bias. Response bias may have interfered with our conclusions, since those who agreed to participate in the study may be in some way different from those who refused to participate. Should all of these potential biases have been avoided it is our conviction that the estimated prevalence of vulnerability would be higher, therefore we believe that despite these limitations this is a valid translation and validation of VES-13.

In our sample, approximately 1 in every 2 elderly cancer patients was identified as vulnerable or frail, which is similar to several published reports (range between 47 and 60 %) [14, 15, 33]. However, persons screened as vulnerable must be carefully evaluated, since the brief VES-13 questionnaire can differ from the longer CGA in identifying some senior patients as vulnerable [34].

## Conclusions

In conclusion, the authors achieved a valid and reliable European Portuguese European version of VES-13, to be used as a first assessment of elderly cancer patients. (Additional file 1). In our clinic, one in two elderly patients was likely to be vulnerable or frail. Therefore a routine clinical practice assessment of the risk of vulnerability, with the use of tools like VES-13, and the development of specialized multidisciplinary teams to perform a comprehensive geriatric assessment, when needed, is paramount if we are to deliver high quality cancer care in an aging population.

## Additional file

Additional file 1: **European Portuguese version of the Vulnerable Elders Survey (VES-13).** (PDF 207 kb)

## References

[1] Howlader N, Noone AM, Krapcho M, Neyman N, Aminou R, Waldron W, et al. SEER Cancer Statistics Review, 1975–2009 (Vintage 2009 Populations), National Cancer Institute. Bethesda, http://issuu.com/ipoporto/docs/ro_nacional_2001?e=7796583/2045184, based on November 2011 SEER data submission, posted to the SEER web site, April 2012.

[2] Portuguese National Oncologic Registry 2001, accessed on December 2011 http://www.ipoporto.min-saude.pt/Downloads_HSA/IPOP/RO_Nacional_2001.pdf

[3] United States Census Bureau. National population projections, accessed on December 2011 http://www.census.gov/population/www/projections; 2008; currently: http://www.census.gov/2010census/

[4] Schrijvers D, Aapro M, Zakotnik B, Audisio R, van Halteren H, Hurria A (2010). ESMO handbook of cancer in the senior patient.

[5] Yancik R (1997). Cancer burden in the aged: an epidemiologic and demographic overview. Cancer.

[6] Extermann M, Hurria A (2007). Comprehensive Geriatric Assessment for Older Patients with Cancer. J Clin Oncol.

[7] Kumar Pal S, Katheria V, Hurria A (2010). Evaluating the Older Patient with Cancer: Understanding Frailty and the Geriatric Assessment. CA Cancer J Clin.

[8] Hurria A, Gupatienta S, Zauderer M, Zuckerman EL, Cohen HJ, Muss H (2005). Developing a Cancer-Specific Geriatric Assessment: A Feasibility Study. Cancer.

[9] Repetto L, Venturi A, Fratino L, Serraino D, Troisi G, Gianni W (2003). Geriatric oncology: a clinical approach to the older patient with cancer. Eur J Cancer.

[10] Hamaker M, Vos A, Smorenburg C, de Rooij S, van Munster B. The Value of Geriatric Assessments in Predicting Treatment Tolerance and All-Cause Mortality in Older Patients with Cancer. Oncologist. 2012;17. 10.1634/theoncologist.2012-0186 PMC3500366 22941970

[11] Puts M, Hardt J, Monette J, Girre V, Springall E, Alibhai S (2012). Use of Geriatric Assessment for Older Adults in the Oncology Setting: A Systematic Review. J Natl Cancer Inst.

[12] Balducci L, Colloca G, Cesari M, Gambassi G (2010). Assessment and treatment of elderly patients with cancer. Surg Oncol.

[13] Saliba D, Elliott M, Rubenstein L, Solomon D, Young R, Kamberg C (2001). The Vulnerable Elders Survey: A tool for identifying vulnerable older people in the community. Journal American Geriatrics Society.

[14] Owusu C, Koroukian S, Schlutchter M, Bakaki P, Berger N (2011). Screening older cancer patients for a Comprehensive Geriatric Assessment: A comparison of three instruments. J Geriatr Oncol.

[15] Luciani A, Ascione G, Bertuzzi C, Marussi D, Codecà C, Di Maria G (2010). Detecting Disabilities in Older Patients With Cancer: Comparison Between Comprehensive Geriatric Assessment and Vulnerable Elders Survey-13. J Clin Oncol.

[16] Kellen E, Bulens P, Deckx L (2010). Identifying an accurate pre-screening tool in geriatric oncology. Crit Rev Oncol Hematol.

[17] Dotan E, Browner I, Hurria A, Denlinger C (2012). Challenges in the management of older patients with colon cancer. J Natl Compr Canc Netw.

[18] Dewolf L, Koler M, Velikova G, Johnson C, Scott N, Bottomley A (2009). EORTC Quality of life group Translation Procedure.

[19] Guillermin F, Bombardier C, Beaton D (1993). Cross-cultural adaptation of health-related quality of life measures: literature review and proposed guidelines. J Clin Epidemiol.

[20] McDowell I (2006). Measuring health : a guide to rating scales and questionnaires.

[21] Nunnally J, Bernstein I (1994). Psychometric Theory.

[22] Streiner DL, Norman GR (1995). Health Measurement Scales - A Practical Guide to Their Development and Use.

[23] The EuroQol Group (1990). EuroQol-a new facility for the measurement of health-related quality of life. Health Policy.

[24] Oken M, Creech R, Tormey D, Horton J, Davis T, McFadden T (1982). Toxicity And Response Criteria Of The Eastern Cooperative Oncology Group. Am J Clin Oncol.

[25] Hanks G, Cherny N, Christakis N, Fallon M, Kasaa S, Portenoy N. Oxford Textbook of Palliative Medicine,4th Edition, Oxford University Press. 1993;109.

[26] Cohen J (1960). A coefficient of agreement for nominal scales. Educ Psychol Meas.

[27] Landis JR, Koch GG (1977). The measurement of observer agreement for categorical data. Biometrics.

[28] Cohen J, Cohen J (1988). Differences between correlation coefficients. Statistical Power Analysis for the Behavioral Sciences.

[29] Pallis A, Fortpied C, Wedding U, Van Nes M, Penninckx B, Ring A (2010). EORTC elderly task force position paper: Approach to the older cancer patient. Eur J Cancer.

[30] Dotan E, Browner I, Hurria A, Denlinger C (2012). Challenges in the management of older patients with colon cancer. J Natl Compr Canc Netw.

[31] Extermann M, Aapro M, Bernabei R, Cohen H, Droz J, Lichtman S (2005). Use of comprehensive geriatric assessment in older cancer patients: Recommendations from the task force on CGA of the International Society of Geriatric Oncology (SIOG). Crit Rev Oncol Hematol.

[32] Hartman J, Forsen J, Wallace M, Neely G (2002). Tutorials in Clinical Research: Part IV: Recognizing and Controlling Bias. Laryngoscope.

[33] Biganzoli L, Boni L, Becheri D, Zafarana E, Biagioni C, Cappadona S (2012). Evaluation of the cardiovascular health study (CHS) instrument and the Vulnerable Elders Survey-13 (VES-13) in elderly cancer patients. Are we still missing the right screening tool?. Ann Oncol.

[34] Molina-Garrido M-J, Guillen-Ponce C (2011). Overvaluation of the vulnerable elders survey-13 as a screening tool for vulnerability. J Clin Oncol.

