# Supplementary material for: Vulnerability in elderly patients with gastrointestinal cancer – translation, cultural adaptation and validation of the European Portuguese version of the Vulnerable Elders Survey (VES-13)
Source: BMC Cancer. 2015 Oct 16;15:723. doi: 10.1186/s12885-015-1739-2 (PMC4609118; doi:10.1186/s12885-015-1739-2)
Supplement: Additional file 1: — European Portuguese version of the Vulnerable Elders Survey (VES-13). (PDF 207 kb) [file 12885_2015_1739_MOESM1_ESM.pdf]

VES-13

1. Idade ______________

Pontuação: 1 ponto para idades entre 75-84 anos
3 pontos para idades ≥ 85 anos

2. Em geral, comparando com outras pessoas da mesma idade, diria que a sua saude é:

- ☐ Má
- ☐ Razoável
- ☐ Boa
- ☐ Muito boa
- ☐ Excelente

Pontuação: 1 ponto para má ou razoável

3. Que dificuldade tem, em média, com as seguintes actividades físicas:

	Nenhuma dificuldade	Pouca dificuldade	Alguma dificuldade	Muita dificuldade	Incapaz de fazer
a. Abaixar-se, agachar-se ou ajoelhar-se? ……………………………………….	☐	☐	☐	☐*	☐*
b. Levantar ou carregar objectos com peso de cerca de 5 kg?...............	☐	☐	☐	☐*	☐*
c. Levantar os braços ou chegar a objectos acima do nível do ombro?	☐	☐	☐	☐*	☐*
d. Escrever, agarrar ou manusear pequenos objectos? ………………………	☐	☐	☐	☐*	☐*
e. Andar 400 metros? …………………………………………………………………………	☐	☐	☐	☐*	☐*
f. Realizar tarefas domésticas pesadas? (pense em tarefas do género de esfregar o chão, lavar janelas ou carregar lenha) ………………………..	☐	☐	☐	☐*	☐*

Pontuação: 1 ponto para cada resposta, de 3a. a 3f. com *
No máximo de 2 pontos

4. Por causa da sua saúde ou condição física, tem alguma dificuldade em:

a. Fazer compras de objectos para uso pessoal? (como higiene pessoal ou medicamentos)

☐ **SIM** → Precisa de ajuda com as compras? ⟶ ☐ SIM * ☐ NÃO

☐ **NÃO**

☐ **NÃO O FAÇO** → Por motivos de saúde? ⟶ ☐ SIM * ☐ NÃO

b. Lidar com dinheiro? (como manter o controlo das despesas ou o pagamento das contas)

☐ **SIM** → Precisa de ajuda para lidar com o seu dinheiro? ⟶ ☐ SIM * ☐ NÃO

☐ **NÃO**

☐ **NÃO O FAÇO** → Por motivos de saúde? ⟶ ☐ SIM * ☐ NÃO

c. Caminhar dentro de casa? (PODE USAR BENGALA OU ANDARILHO)

☐ **SIM** → Precisa de outra ajuda para caminhar? (além da bengala ou andarilho) ⟶ ☐ SIM * ☐ NÃO

☐ **NÃO**

☐ **NÃO O FAÇO** → Por motivos de saúde? ⟶ ☐ SIM * ☐ NÃO

d. Fazer tarefas domésticas leves? (pense em tarefas como lavar a louça, arrumar ou fazer a cama)

☐ **SIM** → Precisa de ajuda com as tarefas domésticas leves? ⟶ ☐ SIM * ☐ NÃO

☐ **NÃO**

☐ **NÃO O FAÇO** → Por motivos de saúde? ⟶ ☐ SIM * ☐ NÃO

e. Tomar banho?

☐ **SIM** → Precisa de ajuda para tomar banho? ⟶ ☐ SIM * ☐ NÃO

☐ **NÃO**

☐ **NÃO O FAÇO** → Por motivos de saúde? ⟶ ☐ SIM * ☐ NÃO

Pontuação: 4 pontos por um ou mais * nas perguntas 4a. a 4e.
